# Lipid Nanocapsule-Chitosan and Iota-Carrageenan Hydrogel Composite for Sustained Hydrophobic Drug Delivery

**DOI:** 10.21203/rs.3.rs-7078136/v1

**Published:** 2025-08-20

**Authors:** Grady K. Mukubwa, Justin B. Safari, Zikhona N. Tetana, Caroline N. Jones, Roderick B. Walker, Rui W.M. Krause

**Affiliations:** Rhodes University; Rhodes University; University of South Africa; The University of Texas at Dallas; Rhodes University; Rhodes University

**Keywords:** Lipid nanocapsules, Hydrogel, Synergistic Use, Drug Delivery, Chitosan, Iotacarrageenan

## Abstract

Hydrophobic drug delivery via oral or pulmonary routes presents significant challenges for clinical translation, particularly for poorly soluble antiviral drugs. Physiological barriers—such as enzymatic degradation, harsh pH, and rapid transit in the gastrointestinal tract, or mucociliary clearance and alveolar macrophage uptake in the lungs—can severely limit therapeutic efficacy. To address these challenges, we developed a novel lipid nanocapsule (LNC) and chitosan/iota-carrageenan hydrogel composite tailored for sustained delivery of hydrophobic antiviral agents. This composite system was designed to encapsulate and deliver Efavirenz (EFV) under simulated gastrointestinal conditions. EFV was first encapsulated in LNCs, which were subsequently embedded within a mucoadhesive hydrogel matrix to form the EFV-LNC hydrogel composite. The LNCs significantly enhanced EFV solubility compared to water alone (p < 0.0001), and droplet size was controlled (57.4 ± 0.5 nm). The hydrogel composite exhibited an optimized swelling ratio (~ 300 g water per 1 g hydrogel) and achieved an encapsulation efficiency of approximately 53%. Importantly, EFV release from the composite was significantly prolonged under various gastrointestinal pH conditions compared to the unformulated drug (p < 0.0001). Cytotoxicity assays confirmed the composite’s cytocompatibility, supporting its potential safety for future mucosal administration. These findings suggest that the LNC-hydrogel composite enhances solubility, enables controlled release, and may improve mucosal retention, supporting its utility as a versatile platform for oral and pulmonary delivery of hydrophobic antiviral drugs.

## INTRODUCTION

Efavirenz (EFV) is a widely prescribed first-line antiretroviral agent belonging to the class of non-nucleoside reverse transcriptase inhibitors (NNRTIs), commonly utilized as part of combination therapy for the treatment of HIV infection^1^. However, it is a highly lipophilic Biopharmaceutics Classification System (BCS) Class II compound with extremely low aqueous solubility (~ 9 μg/mL at 25°C), resulting in dissolution-limited and variable oral absorption (oral bioavailability ~ 40–45%)^1^. This poor and erratic pharmacokinetic profile can lead to inconsistent drug exposure; indeed, genetic variability in EFV metabolism has been linked to subtherapeutic concentrations or doselimiting side effects^2^. These issues pose a significant challenge in maintaining effective and safe EFV plasma levels during longterm HIV treatment. This variability underscores the need for improved EFV formulations to achieve more reliable therapeutic outcomes. Approximately half of all approved antiviral drugs (including lopinavir, ritonavir, and remdesivir) also fall into BCS Class II and suffer from low aqueous solubility^3^. Inadequate dissolution in the gastrointestinal tract is a major factor limiting the efficacy of such oral antivirals, which has motivated the development of formulation strategies to enhance solubility and absorption.

Lipidbased nanoformulations have demonstrated success in improving the oral absorption of BCS II antivirals; for example, ritonavir and saquinavir have been marketed in lipidbased formulations to increase their bioavailability^4–8^. In the case of EFV, various nanocarrier approaches – such as solid lipid nanoparticles, nanoemulsions, and selfemulsifying drug delivery systems – have been explored to boost its dissolution rate and absorption, with encouraging outcomes in preclinical studies^1, 9–11^. These findings reinforce the potential of advanced delivery systems to overcome EFV’s biopharmaceutical limitations. Among lipidbased nanocarriers, lipid nanocapsules (LNCs) are an attractive option for enhancing EFV delivery. LNCs consist of a liquid lipid core surrounded by a surfactant shell, a structure that enables high encapsulation efficiency of hydrophobic drugs like EFV (log P ~ 4.6)^12–16^. The small (~ 20–100 nm) LNC particles disperse readily in gastrointestinal fluids, maintaining EFV in a solubilized state and facilitating its transport to absorptive sites. Encapsulating EFV in LNCs can also convert the drug into an amorphous form, therefore enhancing its apparent solubility^17^. Additionally, LNC formulations help protect the drug from degradation and can modulate its release, which may reduce presystemic metabolism and improve the fraction of EFV absorbed. While LNCs address the solubility issue, a complementary approach is needed to prolong EFV’s residence time in the gut for absorption as LNCs are exposed to rapid gastrointestinal clearance, enzymatic degradation, and premature drug release under harsh gastrointestinal conditions^18–20^.

**Figure 1:**
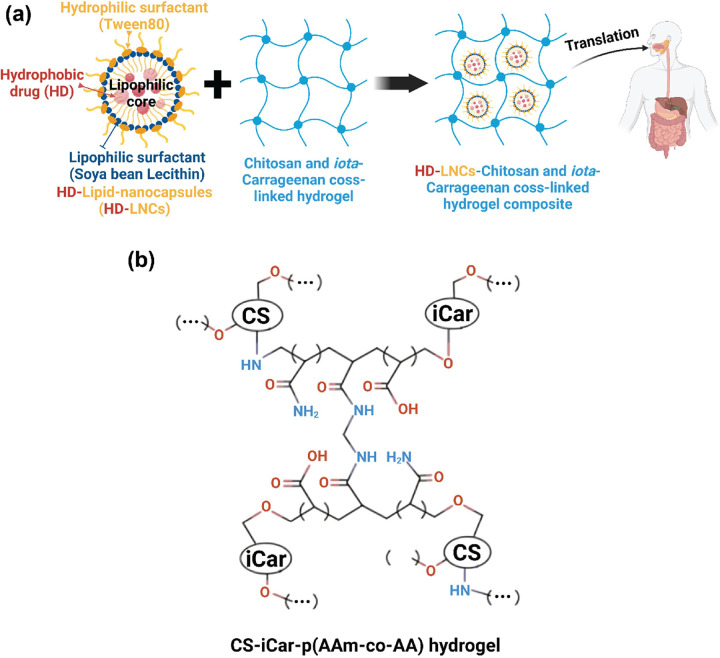
Combination of lipid nanocapsules and chitosan hydrogel for enhanced delivery of hydrophobic drugs and antiviral efficacy. **(a)** The LNCs-hydrogel composite is designed for potential oral delivery of hydrophobic antiviral drugs. This combination leverages the solubility enhancement properties of lipid nanocapsules and the three-dimensional scaffold of the hydrogel, which facilitates better deposition of small particles in the gut regions and their sustained release. Created with BioRender.com. **(b)** Structure of synthesized chitosan and *iota*-Carrageenan crosslinked hydrogel; a detailed chemical reaction of its formation can be found in supporting material **Figure S3**.

In this study, we incorporated EFV-containing lipid nanocapsules (LNCs) into a chitosan/iota-carrageenan hydrogel to create a mucoadhesive nanoparticle-in-hydrogel delivery system (Fig. 1). Chitosan, a cationic polysaccharide, is well known for its mucoadhesive properties, enabling formulations to adhere to the intestinal mucosa and extend drug residence time at the absorption site.^21^. Iota-carrageenan, an anionic polysaccharide, interacts with chitosan to form a crosslinked hydrogel network capable of entrapping the nanocapsules. This biopolymer-based matrix anchors the EFV-containing LNCs in the gastrointestinal environment, limiting rapid transit and enabling sustained drug release. Such hybrid systems combine the solubilization advantages of nanocarriers with the localized retention properties of hydrogels, offering a synergistic strategy to enhance oral drug delivery.^22,23^. We demonstrated that encapsulating EFV in LNCs within a chitosan/iota-carrageenan hydrogel significantly improved key oral biopharmaceutical properties, including solubility, physicochemical stability, sustained release, and cytocompatibility. The LNCs enhanced EFV solubility and protected the drug from premature degradation, while the mucoadhesive hydrogel extended gastrointestinal residence and facilitated more complete absorption. The primary objective of this work was to develop and characterize the LNC-in-hydrogel formulation and to evaluate its potential to overcome EFV’s solubility and delivery limitations. This combined strategy addresses a major formulation challenge by simultaneously improving drug solubility and prolonging mucosal retention. If successful, it could lead to more consistent EFV therapy and serve as a platform for improving the oral delivery of other poorly soluble antiviral agents.

## MATERIAL AND METHODS

This section was adapted from the author’s previously submitted dissertation [Mukubwa, G. K., 2022]^24^.

### Chemicals.

Crude soybean lecithin granules were sourced from Health Connection Wholefoods (USA). As per manufacturer information, the granules predominantly consist of phosphatidylcholine, phosphatidylinositol, polyunsaturated and saturated fatty acids, glycaemic carbohydrates, and sodium. Medium-chain triglyceride (MCT) oil of high purity was acquired from Absolute Organix (Jeppestown, Johannesburg), while Labrafac Lipophile 1349 was kindly provided by Gattfossé (France). Polysorbate 80 (Tween 80) was obtained from Merck (Johannesburg, Gauteng, South Africa), and Efavirenz was generously supplied by Adcock Ingram Ltd. (Wadeville, Gauteng, South Africa)^24^. Additional reagents, including iota-carrageenan (iCar), low molecular weight chitosan (CS), acrylic acid (AA), N,N′-methylenebisacrylamide (MBA), and acrylamide (Aam), were procured from Sigma Aldrich (Darmstadt, Germany). Glacial acetic acid, sodium chloride, and acetone were purchased from Minema (Roodepoort, South Africa), and ammonium persulfate (APS) from Saarchem (Wadeville, South Africa). HPLC-grade acetonitrile was sourced from Merck (Germany), and ultrapure HPLC-grade water was prepared using a RephiLe Bioscience Direct PurefiUltrapure RO Water System (Boston, MA, USA). Human cervical adenocarcinoma (HeLa) cell lines were obtained from ATCC^®^ (Manassas, Virginia, USA)^24^.

### Instruments.

High-performance liquid chromatography (HPLC) was carried out using an Agilent 1100 system comprising a quaternary pump (model G1311A), a degasser (G1322A), a diode array detector (G1315B), and a manual injection unit (G1328B), with separation achieved on a Phenomenex^®^ Kinetex^®^ C18 column (2.6 μm, 100 Å, 150 × 4.6 mm i.d.). Sample lyophilization was performed using a LABCONCO FreeZone^®^ 6 Liter Benchtop Freeze Dryer (Kansas City, MO, USA)^24^. Infrared (IR) spectra were collected using a PerkinElmer Spectrum 100 FTIR spectrometer. Differential scanning calorimetry (DSC) was conducted on a TA Instruments DSC 250. Crystalline structure of the materials was characterized by X-ray diffraction (XRD) using a Bruker D2 Phaser (second generation, Bruker, Billerica, MA, USA). The mean hydrodynamic diameter, polydispersity index (PDI), and zeta potential of the lipid nanocapsules (LNCs) were measured using a Zetasizer Nano ZEN 3600 (Malvern Instruments, Malvern, UK)^24^. Transmission electron microscopy (TEM) was conducted using a Zeiss Libra 120KV instrument (Oberkochen, Germany) to assess particle morphology. Elemental composition was determined via energy-dispersive X-ray spectroscopy (EDS) using an INCA PENTA FET system coupled with a VAGA TESCAM unit (Brno, Czech Republic). Surface features of the hydrogels were examined using a TESCAN VEGA scanning electron microscope (Brno, Czech Republic). Fluorescence imaging was performed with an Olympus BX60 fluorescence microscope (Japan)^24^.

### Preparation of Efavirenz based Lipid Nanocapsules (EFVLNCs).

Efavirenz-loaded lipid nanocapsules were formulated using a phase inversion technique, as established in our earlier work^17^. In summary, the formulation consisted of a mixture of medium-chain triglyceride (MCT) oil, crude soybean lecithin, Tween 80, and aqueous sodium chloride solution (18.5:0.5 g, water:NaCl) in a weight ratio of 12:3:9:76, incorporating Efavirenz at a final concentration of 17.5 mg/mL. Specifically, a total of 25 g of the formulation was prepared by combining 3 g MCT oil, 0.75 g lecithin, 2.25 g Tween 80, and 19 g NaCl solution in a 250 mL round-bottom flask, followed by the addition of 1.35 g of Efavirenz. The mixture was stirred magnetically at 1300 rpm and heated to 95°C using an oil bath on a hot plate. A series of three controlled heating–cooling cycles between 70°C and 95°C were then performed, each lasting approximately 90 minutes, during which phase inversion was visually indicated by the appearance of a translucent emulsion. To induce nanocapsule formation, the emulsion was rapidly quenched by adding cold HPLC-grade water (1–4°C) in a volume three times greater than the initial aqueous phase during the final cycle. A water-cooled condenser was used throughout the procedure to prevent evaporation of volatile constituents.

### Design and Optimization of chitosangiotacarrageenangpoly (acrylamidecoacrylic acid) hydrogel (CS/iCarp(AamcoAA)).

An I-optimal mixture design was conducted using Design-Expert^®^ software version 13 (Stat-Ease, Inc., Minneapolis, MN, USA) to investigate how the composition of the CS/iCar p(AAm-co-AA) hydrogel influences its swelling behavior. The formulation components included two polymeric materials (chitosan and iota-carrageenan), two monomers (acrylic acid and acrylamide), and one crosslinking agent (N,N′-methylenebisacrylamide). These were defined as independent variables, while the hydrogel’s swelling capacity served as the response variable. A polynomial model was fitted to predict the swelling performance based on variations in component proportions. The input variables and their respective percentage contributions, which together sum to 100%, are presented in Table 1.

### Model Optimization and Confirmation.

To evaluate the predictive accuracy of the developed polynomial model, swelling capacity maximization was set as the sole optimization criterion. The software identified the most desirable formulation, which was synthesized in triplicate. Triplicate swelling measurements were then performed, and the resulting average was compared to the model’s 95% prediction interval (95% PI) to validate the model.

(Eq.1) $$95\%\text{PI}={\hat{\bar{y}}}_{0}\pm t_{\left(\frac{\propto }{2}\text{residual}\;df\right)}\times SE_{\text{pred}}$$

where ${\hat{\bar{y}}}_{0}$ is the predicted value of the response, $t_{\left(\frac{\propto }{2}\text{residual}\;df\right)}$ is the Student’s t critical value and *SE*_*pred*_ is the standard error of the prediction^24^.

### Synthesis of CS/iCarp(AamcoAA) hydrogel.

The hydrogel synthesis protocol was adapted from the approach described by Rahmani et al.^25^, with modifications introduced to suit the present study. The hydrogels were fabricated via free radical precipitation graft copolymerization of acrylic acid (AA) and acrylamide (AAm) onto a chitosan–iota carrageenan backbone, using ammonium persulfate (APS) as the radical initiator. For each batch, a total of 500 mg of combined ingredients was prepared. Iota carrageenan was first dissolved in 10 mL of HPLC-grade water at 60°C (Mixture A), while chitosan was separately solubilized in 10 mL of 1% (w/v) acetic acid solution (Mixture B). Both solutions were stirred independently at 800 rpm for 1 hour to ensure uniform dispersion. Subsequently, Mixture B was added to Mixture A, followed by the incorporation of the monomers (acrylic acid and acrylamide) and the crosslinker (MBA) in specified amounts. The polymerization reaction was initiated by adding APS (ranging from 0.05 to 0.2 g) under a nitrogen atmosphere. After polymerization, the hydrogels were thoroughly washed with approximately 500 mL of acetone to remove unreacted residues, and then dried in an oven at 40°C^24^.

### Development of EFVLNCsCS/iCarp(AamcoAA) hydrogel composite.

To develop the EFV LNCs CS/iCar p(AAm-co-AA) hydrogel composite, Efavirenz-loaded lipid nanocapsules were incorporated into the hydrogel matrix, followed by quantification of the entrapped drug. A customized D-optimal design with randomized experimental runs was constructed using Design-Expert^®^ version 13.0 (Stat-Ease, Inc., Minneapolis, MN, USA) to model and optimize the encapsulation efficiency based on varying concentrations of EFV in the lipid nanocapsules. This design yielded 13 experimental runs with EFV concentrations ranging from 17.5 mg/mL to 87.5 mg/mL^24^.

Encapsulation was performed using the swelling equilibrium technique as previously described^26^. In brief, a measured volume of EFV LNCs corresponding to the target EFV concentration was diluted in a 25 mL volumetric flask. This solution was then used to hydrate 50 mg of dried hydrogel for a duration of 24 hours at ambient temperature. After the incubation period, the swollen hydrogel was separated by filtration and rinsed with HPLC-grade water. The filtrate, containing unencapsulated EFV LNCs, was collected for analysis. A 150 μL aliquot of this filtrate was diluted in a 10 mL volumetric flask using a 40:60 acetonitrile–water mixture and analyzed by a reversed-phase HPLC method, as developed by Bienvenu et al. and validated in our previous study^17,27^.

The encapsulation efficiency (EE%) was calculated using the following equation:

(Eq. 2) $$\%\text{EE=}\frac{\text{Mi-Mf}}{\text{Mi}}\times 100$$

Where %EE stands for encapsulation efficiency, Mf is the Efavirenz mass determined from the filtrate and Mi the initial mass of Efavirenz in the soaking solution^24^.

### Droplet size, polydispersity index and zeta potential of EFVLNCs^24^.

Droplet size (DS) and polydispersity index (PDI) measurements were performed using a Malvern Nano ZS Zetasizer (Malvern Instruments, Worcester, UK), operated in dynamic light scattering (DLS) mode for DS/PDI and in laser Doppler anemometry (LDA) mode for zeta potential (ZP) analysis. Before measurement, 500 μL of the saturated nanocapsule suspension was diluted in 50 mL of HPLC-grade water. For DS and PDI assessments, samples were transferred into BRAND^®^ disposable cuvettes (12.5 × 12.5 × 45 mm; BRAND GmbH + CO KG, Wertheim, Germany). Zeta potential determinations were conducted using a folded capillary cell. All measurements were carried out at 22°C with a backscatter detection angle of 173°.

### Swelling Ratio of CS/iCarp(AamcoAA) hydrogel^24^.

The swelling ratio of the synthesized hydrogel, expressed as grams of water absorbed per gram of dry hydrogel (g/g), was determined using a filtration-based approach. A known quantity of dry hydrogel (50–100 mg), denoted as W_0_, was placed into a beaker containing 40–80 mL of HPLC-grade water and allowed to swell over a 24-hour period at room temperature. Subsequently, the mass of a piece of filter paper pre-wetted with distilled water was recorded as W_1_ and placed in a Büchner funnel. The swollen hydrogel was separated from the solution via vacuum filtration to eliminate excess surface water. The filter paper, now holding the swollen hydrogel, was carefully removed and weighed again to obtain the final mass (W_2_). The swelling ratio (SR) was calculated using the equation below, and all measurements were conducted in triplicate for accuracy^28^:

(Eq.3) $$\text{SR(g/g)=}\frac{{\text{W}}_{2}-{\text{W}}_{1}-{\text{W}}_{0}}{{\text{W}}_{0}}\times 100$$

### Fourier Transform InfraRed Spectroscopy (FTIR)^24^.

Fourier-transform infrared (FTIR) spectroscopy was employed to compare the spectral profiles of the synthesized hydrogel and the EFV LNCs–loaded hydrogel composite with those of the individual raw materials, in order to detect potential modifications in functional group characteristics^29^. Spectra were acquired using a PerkinElmer Spectrum 100 FTIR spectrometer operating in attenuated total reflection (ATR) mode^30^. Each sample was analyzed in the spectral range of 650–4000 cm^−1^, with a total of sixteen scans per run^31^. The measurements were performed on freeze-dried forms of both the plain hydrogel and the nanocapsule-loaded composite^30^.

### Thermal Gravimetric Analysis^24^.

Thermogravimetric analysis (TGA) was conducted to evaluate the thermal stability and decomposition patterns of the CS/iCar p(AAm-co-AA) hydrogel and its EFV LNC–loaded composite, in comparison with the native polymers chitosan and iota-carrageenan^32^. The analysis was carried out under an inert nitrogen atmosphere (flow rate: 20 mL/min) using a PerkinElmer TGA 4000 system. For each run, 2–5 mg of sample was heated from 30°C to 600°C at a constant rate of 10°C/min.

### Powder Xray Diffraction^24^.

Powder X-ray diffraction (XRD) was employed to investigate the phase behavior and crystallinity of the freeze-dried CS/iCar p(AAm-co-AA) hydrogel and its EFV LNC–incorporated composite, in comparison with the native polymers chitosan and iota-carrageenan. Crystallinity profiles were analyzed to assess structural differences, and statistical significance in crystallinity variations was evaluated using a one-way ANOVA test. Measurements were conducted using a Bruker D2 Phaser (Second Generation) diffractometer equipped with a LynxEye proportional counter detector, a nickel filter, and Cu Kα radiation (λ = 1.5404 Å). Scans were performed over a 2θ range of 10–60° at a scanning rate of 1°/min, with a fixed slit width of 6.0 mm.

### Scanning Electron Spectroscopy (SEM) and EnergyDispersive Xray Spectroscopy (EDS)^24^.

Scanning electron microscopy (SEM) and energy-dispersive X-ray spectroscopy (EDS) were used to examine the surface morphology and elemental composition of the CS/iCar p(AAm-co-AA) hydrogel and its EFV LNC–loaded composite. The EDS analysis was performed using an INCA PENTA FET system coupled with a VAGA TESCAN unit. Prior to imaging, hydrogels were equilibrated in HPLC-grade water for 24 hours at ambient temperature to reach maximum swelling, then lyophilized using a LABCONCO FreeZone^®^ 6 Liter Benchtop Freeze Dry System. The resulting dried samples were mounted onto aluminum stubs and coated with a thin layer of gold to enhance conductivity before SEM and EDS analysis.

### Transmission Electron Microscopy (TEM)^24^.

Transmission electron microscopy (TEM) was employed to assess the morphology and structural features of the EFV LNCs–loaded CS/iCar p(AAm-co-AA) hydrogel composite at an accelerating voltage of 80 kV. To ensure optimal electron transparency and maintain sample integrity, the composite underwent a carefully controlled preparation protocol. The sample was first embedded in optimal cutting temperature (OCT) compound to provide structural support during sectioning. Ultra-thin sections were then obtained via cryo-ultramicrotomy. These sections were transferred onto 3.05 mm diameter copper grids coated with holey carbon film (FORMVAR/Carbon, 300 mesh; TAAB Laboratories Equipment Ltd., Aldermaston, Berks, UK). For contrast enhancement, the sections were stained with uranyl acetate. Prior to imaging, samples were thoroughly dried in a desiccator to eliminate residual moisture that could interfere with electron beam interaction. This method enabled high-resolution visualization of the hydrogel composite while preserving its native structural characteristics.

### Fluorescence Microscopy^24^.

Fluorescence microscopy was employed to confirm the presence and distribution of lipid nanocapsules (LNCs) within the CS/iCar p(AAm-co-AA) hydrogel matrix. To enable visualization, the composite was stained using amaranth dye. Specifically, 500 μg of amaranth was added to 25 mL of LNCs suspension, and this dye-loaded solution was used to hydrate 50 g of the dried hydrogel, resulting in a stained LNC–hydrogel composite. The composite was subsequently freeze-dried prior to imaging. A small section of the dried sample was placed between two glass slides and mounted under the microscope for observation. Imaging was conducted using an Olympus BX60 fluorescence microscope (Japan), equipped with a U-MWU DM400 filter set for DAPI fluorescence, and images were captured at 10× magnification.

### Drug Release Studies^24^.

The release behavior of Efavirenz (EFV), encapsulated within lipid nanocapsules and further embedded in the CS/iCar p(AAm-co-AA) hydrogel matrix, was evaluated using a 1% sodium lauryl sulfate (SLS) solution as the release medium. Experiments were conducted at two pH levels—7.0 (neutral) and 4.0 (acidic)—to simulate physiological and acidic conditions, respectively. A portion of hydrogel containing an equivalent of 3 mg of EFV was weighed and placed into a dialysis bag (cellulose membrane tubing, flat width 25 mm; Sigma Aldrich, St. Louis, MO, USA), which was then immersed in 25 mL of the release medium. The setup was maintained at 37°C with continuous agitation at 100 rpm. At predetermined intervals (0.5, 1, 1.5, 2, 4, 8, 12, 24, 48, 72, 96, 144, and 216 hours), 5 mL aliquots were withdrawn and replaced with an equal volume of fresh pre-warmed medium to maintain sink conditions. Each time point was analyzed in triplicate. The experiment involved two categorical variables—sampling time and formulation—and one continuous response variable, namely the cumulative percentage of EFV released. It was hypothesized that both the pH of the release medium and the composite nature of the drug delivery system would influence the release kinetics. Two-way ANOVA was employed to assess the statistical significance of these effects across different conditions.

### Cytotoxicity studies^24^.

Cytotoxicity assessments were carried out using HeLa cells. The cells were cultured in Dulbecco’s Modified Eagle Medium (DMEM) supplemented with 10% fetal bovine serum (FBS) and maintained at 37°C in a humidified atmosphere with 5% CO_2_. After a 24-hour incubation period, cells were harvested, resuspended in fresh medium, and seeded into a 96-well plate preloaded with various treatments: unloaded lipid nanocapsules (LNCs), Efavirenz-loaded LNCs (EFV LNCs), LNCs embedded within CS/iCar p(AAm-co-AA) hydrogel, and the fully integrated EFV LNCs–hydrogel composite. All treatments were prepared at a final concentration of 50 μg/mL. The plate was incubated for an additional 24 hours at 37°C and 5% CO_2_ to allow interaction between the cells and the test formulations. Following treatment, resazurin reagent—diluted in culture medium according to manufacturer guidelines (0.1 to 0.5 mg/mL)—was added to each well to cover the cell monolayer. The plate was then returned to the incubator for 1 to 4 hours to allow viable cells to reduce resazurin to fluorescent resorufin. Fluorescence intensity was measured using a multi-well plate reader, with all readings performed in triplicate. Emetine, a known inducer of apoptosis, served as the positive control. Cell viability was calculated using untreated cells as the 100% viability reference, and results were expressed as mean ± standard deviation (SD) based on triplicate measurements. The viability calculation followed a previously reported method^33^.


$$\text{Cell}\;\text{viability}=\frac{\text{Fluo}\;(\text{treated}\;\text{cells})-\text{Fluo}\;(\text{blank})}{\text{Fluo}\;(\text{control})-\text{Fluo}\;(\text{blank})}$$

### Statistical Analysis^24^.

Prior to performing statistical comparisons, data were assessed for normality using the Shapiro–Wilk test. Based on the distribution of the data, appropriate statistical tests were applied. For normally distributed datasets, comparisons between two groups were made using Student’s t-test. When the data did not meet parametric assumptions, the Kruskal–Wallis test was employed as a non-parametric alternative to ANOVA. Post hoc analyses were conducted using Dunn’s test to identify significant pairwise differences, with p-values adjusted for multiple comparisons. Cytotoxicity data involving comparisons between treatment groups and the negative control were analyzed using Welch’s t-test (two-tailed, assuming unequal variances). A p-value of less than 0.05 was considered statistically significant, while results with p < 0.0001 were interpreted as highly significant. Data analysis, modeling, and visualization were carried out using a combination of software tools, including Design-Expert^®^, OriginPro version 9, GraphPad Prism, R, Python, and Microsoft Excel.

## RESULTS AND DISCUSSION

### EFV-lipid nanocapsule preparation and characterization.

EFVLNCs were produced for encapsulation in a chitosan-grafted iota-carrageenan poly(acrylamide-co-acrylic acid) hydrogel (CS/iCar-p(Aam-co-AA)). Solubility tests showed that medium chain triglycerides oil (MCT oil) significantly solubilized Efavirenz compared to water (Ttest, p < 0.0001) (**Figure S1**). The data were normally distributed (Shapiro-Wilk test, p > 0.5) and analyzed using a t-test, revealing with 95% confidence that the true difference in solubility means between the two dissolving media is between 266.9 and 281.4 mg/mL. Based on previous findings reported by Mukubwa *et al*.^17^, an optimized formulation of EFVLNCs was prepared using the phase inversion method^17,34^. The obtained EFVLNCs were characterized for their hydrodynamic diameter, polydispersity index (PDI) and zeta potential (ZP). The average values of these parameters were approximately 57.4 ± 0.5 nm, 0.148, and − 54.9 mV, respectively, consistent with results observed in the previous study^17^.

MCT oil is effective for solubilizing the non-polar EFV molecule in water as it consists of a lipid-structured molecular tail that matches favorably with the hydrophobic moieties present on EFV. This property also makes MCT oil a prime candidate for EFV delivery enhancement. The hydrodynamic diameter of 57.4 nm, a low polydispersity index (0.148), as well as their zeta potential (−54.9 mV) suggests an effective, stable and homogenous nanoparticle system. These properties together indicate that LNCs are effective nanocarriers for drug delivery with enhanced stability and potential enhanced bioavailability^17^.

### Synthesis and Optimization of Swelling Capacity of CS/iCar-p(Aam-co-AA) Hydrogel

The CS/iCar-p(AAm-co-AA) hydrogel was synthesized through free radical copolymerization, with formulation optimization and statistical evaluation performed using Design-Expert^®^ software version 13.0 (Stat-Ease, Inc., Minneapolis, MN, USA). An I-optimal mixture design yielded 35 distinct formulations, each prepared according to the synthesis protocol described earlier. **Table S1** summarizes the component ratios for each formulation alongside their corresponding swelling capacities, which were used as the response variable for optimization. Swelling capacity was expressed as a percentage, calculated by the ratio of water absorbed (g) to the dry hydrogel mass (g). The proposed reaction mechanism for hydrogel formation is depicted in **Figures S2 and S3**. Multiple polynomial models were evaluated to fit the experimental data, with the linear model demonstrating superior performance. This model yielded consistently low standard errors (< 0.5σ, where σ denotes the estimated variability within the dataset) across the design space (**Figure S4**). **Table S2** presents a comparative analysis of model fits, where the linear model was automatically selected as optimal based on its lowest predicted residual error sum of squares (PRESS) value and a significantly small sequential p-value, both indicators of strong model predictive accuracy^11^.

While swelling behavior was selected as the primary criterion for optimizing the CS/iCar-p(Aam-co-AA) hydrogel, given its direct relationship with drug absorption and controlled release in gastrointestinal conditions, rheological properties also critically influence the composite’s performance. Mechanical and rheological characteristics significantly impact hydrogel behavior within the dynamic gastrointestinal environment, affecting aspects such as mucoadhesion, retention time, and stability against physiological shear forces. Evaluating rheological parameters, including viscosity, elasticity, and shear-thinning behavior, is therefore crucial to predict the hydrogel’s performance under realistic gastrointestinal conditions^35,36^. Although detailed rheological characterization was beyond the scope of this study, future work should investigate these properties to optimize the formulation further, ensuring enhanced gastrointestinal retention, controlled drug release, and improved patient compliance in oral antiviral drug delivery applications.

### Varying the portion of polymers, monomer and crosslinkers either increased or decreased the swelling capacity.

In this study, a linear regression model was developed to accurately predict the swelling ratio of hydrogels based on the proportions of iota-carrageenan, chitosan, acrylic acid, acrylamide, and N,N′-methylenebisacrylamide (MBA) used in the formulation. These five components served as the independent variables in the model. The resulting predictive equation for swelling behavior is given as:

(Eq. 4) %SC=0.556464×A+−0.241395×B+0.0694467×C+0.0907205XD+0.0431736XE

In the regression model, factors A through E represent the following components: iota-carrageenan (A), chitosan (B), acrylic acid (C), acrylamide (D), and N,N′-methylenebisacrylamide (E).

This model is statistically significant (p = 0.0451), but it only accounts for a moderate portion of the variance in swelling ratio (adjusted R^2^ = 0.1643). The insignificant lack-of-fit p-value (0.3168) means that this model is decent for predicting data without being an overfit and thus serves as a good reference to formulate hydrogels in the future^37–39^.

The low R^2^ value is likely to be due to variability in hydrogel synthesis; small differences during preparation can cause significant variations in swelling behavior^40,41^. While the model is linear and insubstantial in as much as mimicking more complex component interactions, it provides a useful approximation of swelling behavior within these experimental confines. More complex models could improve prediction, but this simple linear regression provides a nice balance between simplicity and accuracy for early-stage hydrogel development.

**Figure 2:**
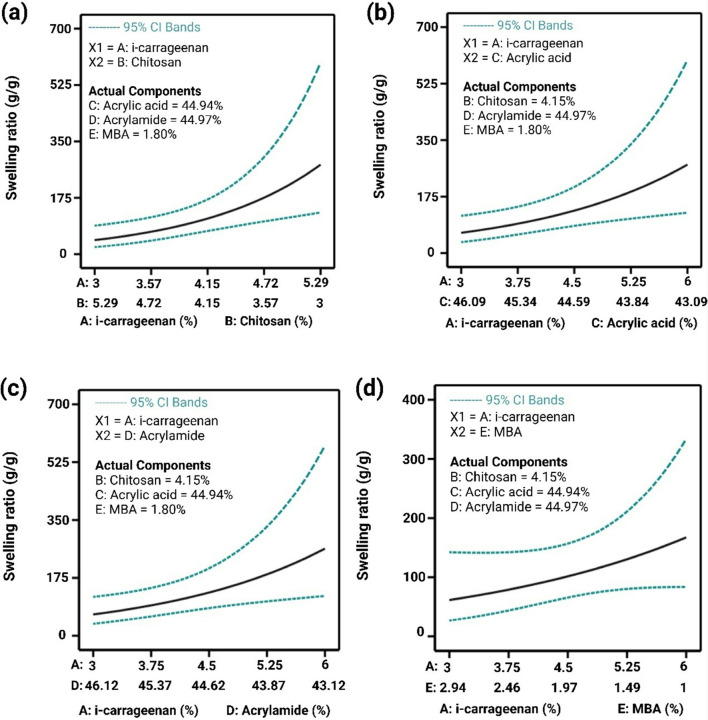
Controlling the swelling capacity of hydrogels by varying proportions of monomers, polymers, and crosslinker. **(a)** A combination of high iota-carrageenan content with reduced chitosan levels significantly enhances the swelling behavior of the hydrogel; **(b)** Increasing iota-carrageenan while simultaneously lowering the amount of acrylic acid leads to greater swelling capacity; **(c)** A higher proportion of iota-carrageenan paired with a lower proportion of acrylamide results in increased water absorption; and **(d)** Swelling capacity also increases when iota-carrageenan content is elevated while the concentration of N,N′-methylenebisacrylamide (MBA) is reduced.

The swelling ratios of the synthesized hydrogel formulations varied significantly, ranging from 2.84 to 434.43. This variation was directly influenced by the relative composition of the hydrogel constituents. An increase in iota-carrageenan content led to a marked enhancement in swelling capacity, whereas higher chitosan levels were associated with a reduction in water absorption (Fig. 2). Additionally, decreasing the proportions of acrylic acid, acrylamide, and MBA resulted in improved swelling performance. This trend is attributed to lower crosslinking density at reduced monomer and crosslinker concentrations, which facilitates greater expansion of the hydrogel network and allows more water uptake.

### Validation of the hydrogel composition model yielded a swelling ratio exceeding 300, meaning that over 300 g of water was absorbed per gram of hydrogel.

**Figure 3:**
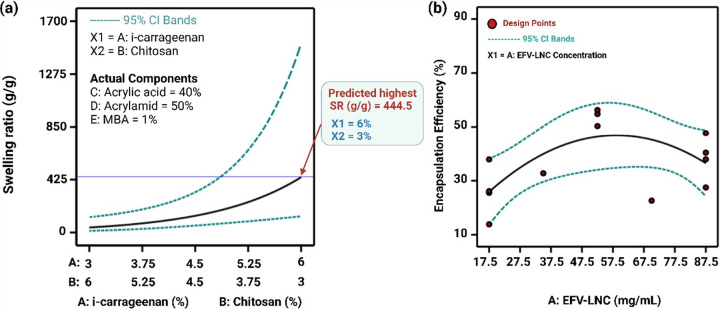
Optimal swelling capacity of CS/iCar p(Aam-*co*-AA) hydrogel and encapsulation efficiency of EVF-LNCs in CS/iCar-p(Aam-co-AA) hydrogel. (a) Highest and optimal swelling capacity (%SC) with corresponding proportions. Of the starting materials used for the rest of the experiments, namely the encapsulation capacity and drug release study. (b) Eight different concentrations of encapsulated EFV in LNCs were evaluated to yield the optimized hybrid system with optimum EVF concentration. The curve shows that the encapsulation efficiency stops increasing after reaching concentrations between 46 – 55 mg/mL.

To assess the predictive accuracy of the developed linear regression model, Design-Expert^®^ software generated five optimized formulations based on the criterion of maximizing swelling capacity. Among these, the formulation with the highest desirability score was selected for experimental validation and tested in triplicate, as summarized in **Table S3**. The optimized formulation consisted of 6% iota-carrageenan, 3% chitosan, 40% acrylic acid, 50% acrylamide, and 1% MBA, as also depicted in Fig. 3a. The average swelling ratio observed experimentally fell within the 95% prediction interval, thereby confirming the model’s reliability.

The close overlap of observed and predicted swelling ratio values for the 95% prediction interval (Table 2), confirms the effectiveness of our prediction model. This predictability indicates that the model can reasonably estimate swelling ratios despite inherent variability in hydrogel synthesis. This inherent variability is reflected in the wide prediction interval of the swelling ratios provided by the model (32.6 to 1407.1). Overall, this model can be used to predict different proportions of iCar, CS, AA, Aam and MBA to synthesize a hydrogel with desired properties. It also underlines the potential of our optimized hydrogel formulation in drug delivery systems where maximum swelling is required. This formulation was designated for further experiments, including LNCshydrogel composite preparation, EFVLNCshydrogel composite preparation and EFV release studies.

### CS/iCarp(AamcoAA) hydrogel composite entrapped LNCs containing over 50 mg/mL of EFV^24^.

Following the synthesis of the CS/iCar p(AAm-co-AA) hydrogel, an Efavirenz-loaded LNC solution was incorporated to form the EFV LNCs–hydrogel composite. To investigate the impact of LNC drug concentration on encapsulation efficiency, thirteen different formulations were prepared based on a D-optimal (custom) randomized experimental design (Fig. 3). A clear trend emerged wherein encapsulation efficiency increased with rising EFV concentrations, reaching a broad maximum between 55 and 60 mg/mL before declining at higher concentrations. The experimental data were modeled using a reduced quadratic equation, as outlined below:

(Eq. 5) $$\%\text{EE}=4.25921+1.4527\text{t}1\times \text{A}+-0.0123934\times {\text{A}}^{2}$$

where A represents the concentration of Efavirenz (EFV) within the lipid nanocapsules (LNCs).

To identify an optimal EFV LNC concentration for subsequent experiments and to evaluate the model’s predictive capability, encapsulation efficiency was set as the primary optimization target. Design-Expert^®^ software generated a single optimal formulation, a concentration of 58 mg/mL EFV LNCs was recommended, corresponding to a predicted encapsulation efficiency of 46.83%. In practice, 50 mg of the optimized hydrogel were immersed in 25 mL of an EFV LNC solution containing 58.61 mg/mL of the drug. The experimentally determined encapsulation efficiency fell within the 95% prediction interval (Table 3), confirming the model’s validity for predictive applications.

### The CS/iCar p(AAm-co-AA) hydrogel exhibited thermal stability above 200°C.

**Figure 4:**
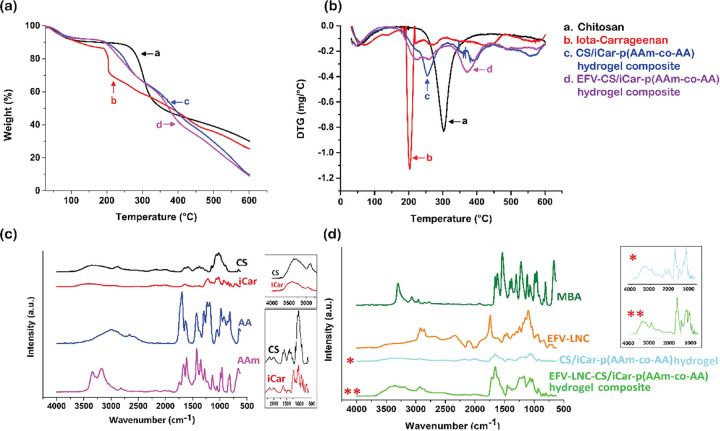
Thermal gravimetric and fourier transform infrared spectroscopy analysis of hydrogels. (a) Thermogravimetric analysis (TGA) profiles showing thermal decomposition of CS/iCar p(AAm-co-AA) hydrogel, EFV LNCs–loaded CS/iCar p(AAm-co-AA) hydrogel composite, chitosan (CS), and iota-carrageenan (iCar), with weight loss plotted against temperature. (b) First-order derivative curves (DTG) of the TGA data illustrating the rate of decomposition. (c) FTIR spectra of the raw materials: chitosan (CS), iota-carrageenan (iCar), acrylic acid (AA), and acrylamide (AAm). (d) FTIR spectra of N,N′-methylenebisacrylamide (MBA), Efavirenz-loaded lipid nanocapsules (EFV-LNCs), CS/iCar p(AAm-co-AA) hydrogel, and the EFV-LNCs-CS/iCar p(Aam-co-AA) hydrogel composite.

Thermogravimetric analysis (TGA), conducted to compare the thermal behavior of the synthesized hydrogel with its individual components, revealed a series of distinct decomposition events corresponding to the chemical crosslinking process. The incorporation of acrylic acid and acrylamide into the polysaccharide network altered the degradation profile of the final hydrogel. As shown in Fig. 4a, multiple weight loss stages were observed during thermal decomposition. Among the base polysaccharides, iota-carrageenan demonstrated a more rapid thermal degradation, with approximately 28% weight loss at 208°C, whereas chitosan showed only a 10% reduction at the same temperature. Figures 4a and 4b illustrate the unique decomposition pattern of the hydrogel, confirming its enhanced thermal resistance beyond 200°C. These findings support the successful chemical modification of the polysaccharide matrix, as reflected in its altered thermal characteristics.

The CS/iCarp(AamcoAA) hydrogel demonstrated excellent thermal stability, exceeding 200°C, due to chemical crosslinking that improved its resistance to thermal degradation. The decomposition profiles in Figs. 4a and 4b illustrate that chitosan and iota-carrageenan undergo distinct degradation patterns, indicating their unique contributions to the composite’s thermal resilience. Such thermal stability is particularly advantageous for oral drug delivery formulations, as it ensures that manufacturing processes involving elevated temperatures (e.g., drying, tablet compression, sterilization) will not compromise hydrogel integrity^42–44^. Additionally, these properties suggest the hydrogel’s suitability for other advanced biomedical applications, such as transdermal patches or implantable devices requiring sterilization via autoclaving^45^. These observations reinforce the importance of chemical crosslinking for obtaining hydrogels with robust thermal stability suitable for various biomedical applications, including oral dosage forms^46^.

### Chemical interactions between polymers, monomers, and the crosslinker resulted in shifts in wavenumbers observed in the FTIR spectra of the synthesized hydrogel.

Fourier-transform infrared (FTIR) spectroscopy was utilized to examine potential chemical bonding and structural interactions within the material resulting from the synthesis of the CS/iCarp(AAmcoAA) hydrogel. Both chitosan (CS) and iota-carrageenan (iCar) contain hydroxyl groups that may interact with the carbon–carbon double bonds (−C = C−) present in the monomers and crosslinker. Additionally, interactions between the EFV-loaded lipid nanocapsules (EFV LNCs) and the hydrogel network were evaluated through spectral analysis. FTIR spectra of the synthesized materials were compared with those of the raw components (Figs. 4c and 4d). The hydrogel spectrum displayed a broad absorption band in the 3300–3500 cm^−1^ range, corresponding to O–H and N–H stretching vibrations. Peaks between 1000 and 1200 cm^−1^ were attributed to C–O stretching, while weaker bands observed in the 2000–2300 cm^−1^ region, also present in CS and iCar, were likely overtones of C–O vibrations, possibly arising due to the thickness of the hydrogel or polysaccharide films. The prominent peak at 1657 cm^−1^ confirmed the presence of C = O groups, consistent with the incorporation of acrylamide and acrylic acid. The FTIR spectrum of the EFV LNCs–loaded hydrogel composite retained the key absorption bands observed in the plain hydrogel. However, the 2000–2300 cm^−1^ bands appeared diminished or absent, potentially due to differences in film thickness. Notably, additional bands in the 2950–2840 cm^−1^ range, characteristic of aliphatic C–H stretching from saturated fatty acids, were evident in the composite, along with carbonyl peaks near 1750–1745 cm^−1^, consistent with the presence of lipid-based nanocapsules.

The wavenumber shifts that we observed in the FTIR spectra following CS/iCarp(AamcoAA) hydrogel and its EFV-LNCs composite synthesis demonstrates successful yield of a stable hydrogel network from chemical interactions among the polymers, monomers, and crosslinking agent. The acrylamide and acrylic acid grafting onto the polysaccharide backbone is confirmed by the presence of stretches like the OH and NH, coupled with C = O and CO vibrations, aligning with previous studies where similar spectral shifts have been demonstrated when polysaccharides are chemically modified to enhance their drug delivery potential^47,48^. For example, improvement of properties like the swelling ratio have been proven to be associated with the introduction of functional groups like amides and carboxylates in the hydrogel’s structure^49^. Moreover, upon EFVLNCs incorporation, the hydrogel structure was maintained, suggesting that the encapsulation process does not compromise the hydrogel’s integrity. On the other hand, the presence of CH bands which are characteristic of saturated fatty acids found in medium chain triglycerides oil (MCT oil), supports successful encapsulation of EFV-LNCs^50^. Overall, this new CS/iCarp(AamcoAA) hydrogel delivery system possesses potential practical applications in improving controlled drug delivery of hydrophobic drugs like EFV.

### The synthesized CS/iCar p(AAm-co-AA) hydrogel displayed a fibrous surface architecture, with elemental analysis revealing the presence of nitrogen and sulfur groups indicative of its polysaccharide components.

**Figure 5:**
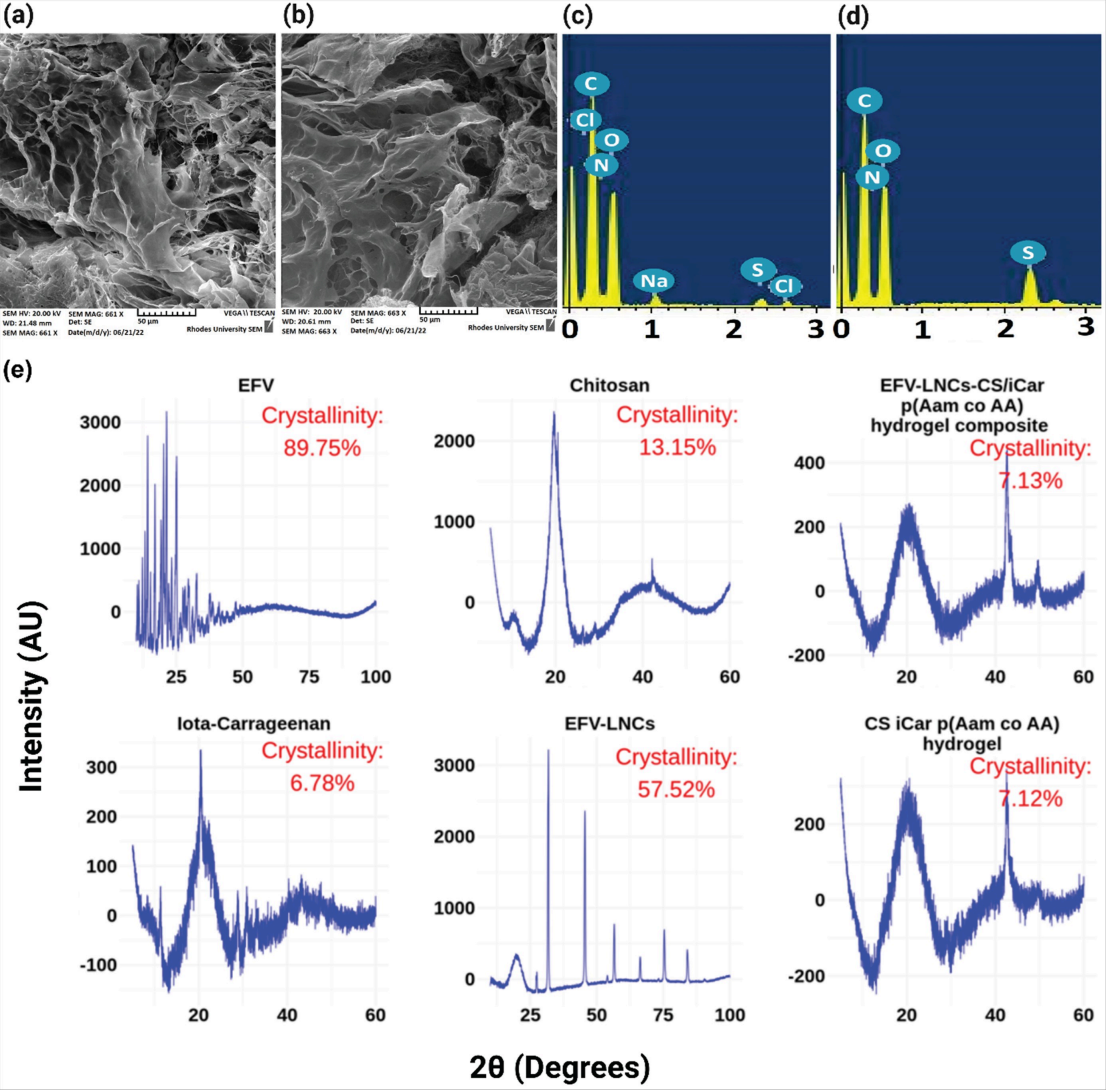
Nitrogen and sulfur appeared on the surface of porous CS/iCar-p(Aam-co-AA) hydrogel structure and crystallinity significantly changes during hydrogel synthesis and addition of EFV. Scanning electron micrographs (scale bar: 50 μm) reveal the porous surface morphology of **(a)** the unloaded CS/iCar p(AAm-co-AA) hydrogel and **(b)** the EFV LNCs–loaded composite hydrogel. Energy-dispersive X-ray spectroscopy (EDS) spectra indicating the elemental composition of **(c)** the EFV-LNCs-CS/iCar p(AAm-co-AA) hydrogel composite and **(d)** the unloaded hydrogel, confirming the presence of nitrogen, sulfur, carbon, oxygen, and chlorine attributed to Efavirenz. **(e)** Powder X-ray diffraction (XRD) patterns comparing the crystallinity profiles of the CS/iCar p(AAm-co-AA) hydrogel and its EFV LNCs composite with the raw materials—chitosan, iota-carrageenan, free Efavirenz (EFV), and EFV-LNCs—alongside their corresponding mean crystallinity values.

Surface morphology and elemental composition were evaluated using scanning electron microscopy (SEM) and energy-dispersive X-ray spectroscopy (EDS), respectively. SEM micrographs (Fig. 5a: EFV LNC–loaded hydrogel composite; Fig. 5b: unloaded hydrogel) revealed a rough, fibrous network with abundant porous structures, which are potentially advantageous for drug loading and release^51^. The incorporation of EFV LNCs did not visibly alter the porous morphology of the hydrogel. EDS spectra further confirmed the elemental signatures of the hydrogel matrix. Both the composite (Fig. 5c) and unloaded hydrogel (Fig. 5d) exhibited characteristic peaks for nitrogen, originating from chitosan and sulfur, derived from iota-carrageenan, alongside common elements such as carbon and oxygen. Additionally, chlorine was detected exclusively in the EFV-loaded composite (Fig. 5c), corresponding to the chemical structure of Efavirenz.

These results indicate that both CS/iCar-p(Aam-co-AA) hydrogel and its EFV-LNCs composite exhibit porous and fibrous structures suitable for drug delivery. Although pore size was not determined, the previously observed correlation between the swelling ratio and the proportion of the hydrogel components implies that pore sizes also vary accordingly. For instance, increasing the amount of iota-carrageenan and decreasing the amount of chitosan resulted in higher swelling ratios, implying the presence of larger or more accessible pores, with the potential to affect drug loading and release. In the future, pore size should be measured and optimized to further understand its relationship with swelling ratio, drug loading and drug release^52–55^.

### The CS/iCarp(AamcoAA) hydrogel and EFVLNCsCS/iCarp(AamcoAA) hydrogel composite display relatively lower crystallinity compared to chitosan and iota-carrageenan.

Powder X-ray diffraction (XRD) analysis was performed to evaluate changes in the crystalline structure following hydrogel formation and drug encapsulation. The data passed normality testing using the Shapiro–Wilk test (p > 0.05), and subsequent ANOVA analysis revealed that the crystallinity of both the plain hydrogel and the EFV-loaded composite was significantly lower than that of chitosan, EFV, and EFV-LNCs (p < 0.01), as shown in Fig. 5e. These findings provide additional evidence of successful chemical crosslinking between the monomers and polysaccharides. Crosslinking typically disrupts the orderly molecular arrangement in a polymer matrix, thereby reducing its degree of crystallinity^56^.

The crystallinity reduction of Efavirenz upon encapsulation, first from 89.75% for nonencapsulated EFV to 57.52% for EFV-LNCs, and further down to 7.13% for the EFVLNCsCS/iCarp(AamcoAA) hydrogel composite highlights the effectiveness of double encapsulation in influencing the drug’s polymorphism. Interestingly, CS/iCarp(Aam-co-AA) hydrogel’s crystallinity which was initially 7.12% was not affected by EFV-LNCs incorporation. This supports that CS/iCar-p(Aam-co-AA) hydrogel’s structural integrity was maintained. Decreasing EFV crystallinity is critical since lower crystallinity improves solubility and bioavailability, which are essential for effective drug delivery. Moreover, CS/iCar-p(Aam-co-AA) hydrogel structural integrity maintenance suggests that its matrix can entrap a drug without altering its inherent properties.

Our previous Differential Calorimetric Analysis (DSC) of EFV-LNCs demonstrated the transformation of encapsulated EFV to an amorphous state, explaining the reduction of its crystallinity rather than mere masking it^17^. DSC analysis of the EFVLNCsCS/iCarp(AamcoAA) hydrogel composite compared to non-encapsulated EFV should be preconized to further demonstrate how the hydrogel matrix affected EFV polymorphism. When compared to Chitosan, CS/iCarp(AamcoAA) hydrogel crystallinity significantly decreased, and this could be accounted for by the introduction of a threedimensional network that limits the mobility of Chitosan resulting in a more amorphous structure^57^.

### The microstructural insights of EFVLNCsCS/iCarp(AamcoAA) hydrogel composite reveal generated entanglements of the hydrogel scaffold and tiny spherical particles of entrapped EFVLNCs.

**Figure 6:**
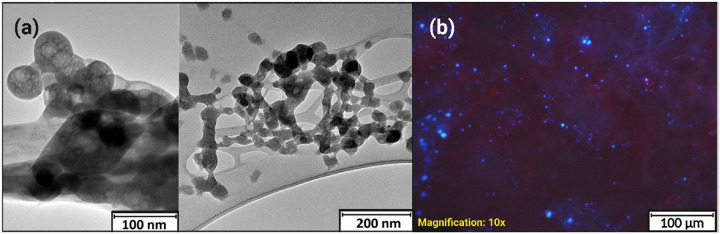
TEM and fluorescent microscopy analysis of EFV-LNCs-CS/iCar-p(Aam-co-AA) hydrogel composite. **(a)** TEM micrographs of EFV-LNCs-CS/iCar-p(Aam-co-AA) hydrogel composite highlighting scaffold entanglements and tiny particles at 100 nm (left) and 200 nm (right), respectively; **(b)** Micrograph of LNCs-CS/iCar-p(Aam-co-AA) hydrogel composite visualized under fluorescence microscope using U-MWU DM400 filter (DAPI fluorescence) at 10x magnification, the fluorescent amaranth purple dyed dots being indicative of LNCs clusters embedded in hydrogel at 10x magnification.

Transmission electron microscopy enabled the visualization of the microscopic structure of the composites. Figure 6a highlights the EFV LNCs CS/iCar p(Aam-co-AA) hydrogel composite network, showing proposed entanglements at 200 nm. At 100 nm, small spherical particles are visible, indicating the presence of EFV LNCs. A TEM micrograph of EFVLNCs has previously been reported, exhibiting particles with sizes below 100 nm^17^. The presence of these particles confirms that the EFVLNCs particles are incorporated into the hydrogel matrix, and their distinct appearance suggests a specific and controlled distribution. This insight into the composite structure further supports the entrapment of EFVLNCs in the hydrogel scaffold. This entrapment could have important implications for drug delivery, where the controlled release of the encapsulated EFV is desired.

### EFVLNCs particles entrapped in the CS/iCarp(AamcoAA hydrogel scaffold

The presence of the EFV-LNCs in CS/iCar p(Aam-co-AA) hydrogel scaffold was confirmed by using fluorescence microscopy since the LNCs lacked a heavy atom. Amaranth dye was used to stain the composite to increase the contrast between the incorporated LNCs and the rest of the constituents in the composite. The images obtained using the DAPI filter (Fig. 6b), which excites at 358 nm and emits blue fluorescence at 461 nm, revealed a bright reddish color of the background corresponding to the outer hydrogel layer. Besides, the bright and distinct blue-color dots represent the clusters of LNCs. While Amaranth dye is weakly fluorescent, its association with the phospholipids in soybean lecithin of the LNCs likely enhanced the fluorescent signal. This is consistent with previous research on soybean oil and soy protein, where phospholipid interactions with some molecules resulted in amplified fluorescence under specific conditions^58^. The blue fluorescence not only evidenced the structural integrity of the EFV-LNCs-CS/iCar p(Aam-co-AA) hydrogel composite but also the success of the encapsulation procedure, making a huge potential for further application, including controlled release of the entrapped drug (EFV).

### LNCsCS/iCarp(AamcoAA) hydrogel composite achieves enhanced and sustained In vitro Release of EFV.

**Figure 7:**
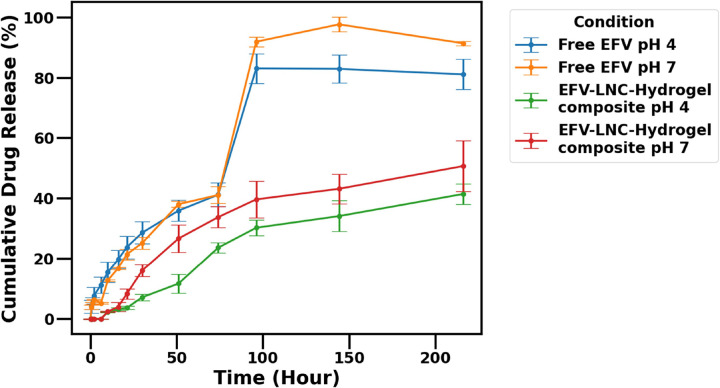
EFV-LNCs-CS/iCar-p(Aam-co-AA hydrogel composite prolongs release of drug at both pH 4 and 7. Cumulative release profiles of Efavirenz encapsulated in the hydrogel composite at pH 7 and pH 4, demonstrating prolonged release of the encapsulated EFV (red and green lines) compared to the non-encapsulated EFV (orange and blue lines). Wilcoxon signed-rank test revealed no significant difference between Free EFV at pH 4 and pH 7 (n.s., p = 0.807), while encapsulation significantly slowed release in both conditions (p ≤ 0.001). Furthermore, the hydrogel composite demonstrated pH-responsive release behavior, with significantly greater Efavirenz release observed at pH 7 compared to pH 4 (p = 0.0077). These results underscore the composite’s capacity for controlled and sustained drug delivery, highlighting its potential for targeted release under physiological conditions. n.s. = not significant; *** = p ≤ 0.001. Experiments were conducted in triplicate.

The in vitro release study was conducted using a 1% sodium lauryl sulfate (SLS) solution adjusted to pH 7 and pH 4 to simulate gastrointestinal conditions, providing physiologically relevant environments for assessing drug release. The pH 7 condition represents the intestinal environment, while pH 4 condition simulates fed-state gastric conditions or the transitional environment between the stomach and proximal intestine, conditions frequently experienced after food intake, thereby enabling evaluation of the composite’s performance throughout different regions of the gastrointestinal tract^59–61^. Figure 7 illustrates the comparative release profiles of free Efavirenz (non-encapsulated) and the EFV LNCs–CS/iCar p(AAm-co-AA) hydrogel composite under the tested conditions. As the data did not meet normality assumptions (Shapiro–Wilk test, p < 0.05 for all groups), the Kruskal–Wallis test was employed for non-parametric analysis of overall group differences. This was followed by Dunn’s post-hoc test for pairwise comparisons. The Kruskal–Wallis test identified statistically significant differences in the drug release behavior between free EFV and the hydrogel composite across multiple time points. Free EFV exhibited a significantly faster release, exceeding 75% in both pH conditions over the 9-days experimental period (p < 0.0001). Notably, the free EFV release profile demonstrated a burst increase after 72 hours (region between 50 ~ 100 hours as seen in Fig. 7), likely due to saturation of the dialysis bag, alterations in solution properties, and the lack of a diffusion barrier, all of which contribute to unrestricted drug diffusion. In other words, EFV saturated the dialysis bag and precipitated; once some precipitated drug gradually dissolved or the medium was refreshed, a sudden increase in diffusion occurred^62^. In contrast, the hydrogel formulation averted such bursts, maintaining a steadier, more linear release without spikes.

Pairwise comparisons were examined using the Wilcoxon Signed-Rank Test. The release kinetics of Free EFV at pH 4 and pH 7 were not significantly different (W = 42.0, p = 0.807), suggesting that pH had minimal influence on free EFV diffusion dynamics. However, significant differences were observed between Free EFV at pH 4 vs. EFVLNCCS/iCar at pH 4 (W = 0.0, p = 0.0015) and Free EFV at pH 7 vs. EFVLNCCS/iCar at pH 7 (W = 0.0, p = 0.0015), confirming that encapsulation significantly reduced drug release rates under both pH conditions. Additional comparisons between Free EFV at pH 4 vs. EFVLNCCS/iCar at pH 7 (W = 0.0, p = 0.0015) and Free EFV at pH 7 vs. EFV-LNC-CS/iCar at pH 4 (W = 0.0, p = 0.0015) further reinforced the hydrogel’s ability to modulate drug release more effectively than the free drug, irrespective of pH. The composite’s sustained, slower release is governed by diffusion through multiple barriers and pH-responsive matrix behavior. EFV must traverse the lipid nanocapsule core and then the chitosan/iota-carrageenan hydrogel network, which slows down its mobility^63^.

The hydrogel’s mesh size and swelling depend on pH – it stays dense at pH 4 (limiting EFV diffusion) and expands at pH 7 (allowing faster diffusion)^64^. Interestingly, a significant difference was detected within the EFV-LNC-CS/iCar hydrogel composite, with higher drug release at pH 7 compared to pH 4 (W = 0.0, p = 0.0077). This pH-dependent release profile suggests that the hydrogel matrix responds to environmental pH, releasing less drug under mildly acidic conditions. This means that there is minimal release in the stomach (pH 4) means most EFV remains protected from acidic degradation or irritation, and the dose is not wasted where little drug uptake occurs. Once the hydrogel reaches the intestine and encounters pH 7, it swells and rapidly releases EFV at the site of optimal absorption^65^. This targeted release maximizes drug bioavailability by delivering EFV where it can be efficiently absorbed, while the sustained release profile prolongs drug presence in the absorption window. Given that pH 4 simulates fed-state gastric conditions or the transitional environment between the stomach and proximal intestine, this characteristic could be advantageous for protecting the drug from premature release in the stomach and promoting more targeted and sustained drug release upon entering the neutral intestinal environment, ultimately enhancing oral bioavailability and therapeutic efficacy as already demonstrated in previous studies^66,67^.

The hydrogel composite demonstrated sustained and controlled drug release over 72 hours without significant burst effects, in contrast to free EFV. The hydrogel’s porous structure effectively acted as a diffusion barrier, modulating EFV release through swelling and erosion under gastrointestinal conditions. This controlled release profile is advantageous for improving EFV’s oral bioavailability and reducing dosing frequency. LNCs enhance EFV’s solubility and stability, preventing precipitation and protecting the drug from premature degradation, while also facilitating absorption via mechanisms like lymphatic uptake^68^. The hydrogel, in turn, localizes and slowly releases the LNCs (and encapsulated EFV) in the GI tract. This dual system offers a synergistic effect that prevents the rapid clearance or burst release that might occur with LNCs alone and effectively marries the sustained release capacity of hydrogels with the absorption-enhancing benefits of nanoparticles^69^. Consequently, the composite facilitates prolonged drug retention and absorption compared to either component alone, highlighting its potential as an advanced oral delivery platform to enhance therapeutic efficacy and patient compliance for poorly soluble antiviral drugs.

### Hydrogel is biocompatible with HELA cells.

Cytocompatibility is essential to ensure future clinical use of any newly developed product. For this reason, we performed cytotoxicity experiments by treating HeLa cells with lipid nanocapsules, CS/iCarp(AamcoAA) hydrogel, LNCsCS/iCarp(AamcoAA) hydrogel composite and EFVLNCsCS/iCarp(AamcoAA) hydrogel composite using a resazurinbased assay. HeLa cells were chosen due to their widespread use and well-characterized response in preliminary cytotoxicity studies. This allows for consistency and comparability with existing literature, providing a reliable initial assessment of biocompatibility^70–73^. The treatments were prepared in a 96 well plate at a concentration of 50 μg/mL. The plate was put in the incubator at 37°C with 5% CO_2_ to allow the cells to grow and interact with the treatments for 24 hours. After incubation, the resazurin was used to fully cover each well of the 96-well plate, which was then returned into the incubator. In this resazurin-based assay, viable cells with active metabolism reduce the non-fluorescent resazurin to the fluorescent compound resorufin. The resulting fluorescence intensity, measured after 1–4 hours of incubation, is directly proportional to the number of metabolically active cells. Cytotoxicity was expressed as percent viability, calculated as the ratio of fluorescence in wells with treated cells compared to wells containing untreated cells.

**Figure 8:**
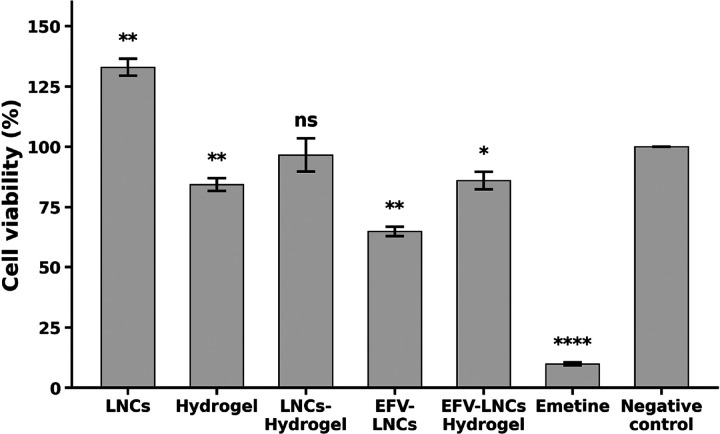
New LNCs-hydrogel composite is cytocompatible. Viability of HeLa cells treated with various formulations-LNCs [50 μg/mL], Hydrogel [50 μg/mL], LNCs-Hydrogel composite [50 μg/mL], EFV-NCs, EFV-LNCs Hydrogel composite, and Emetine [50μg/mL] (positive control)—compared to a Negative control (untreated cells), assessed using the resazurin assay. LNCs significantly increased viability(~135%, t = 16.1692, p = 0.0038, **), while Hydrogel (~85%, t = −10.1806, p = 0.0095, **), EFV-LNCs(~65%, t = −30.7915, p = 0.0011, **), and EFV-LNCs Hydrogel (~85%, t = −6.7239, p = 0.0214, *) significantly reduced viability. The LNCs-Hydrogel composite (~90%, t = −0.8552, p = 0.4825, ns) did not significantly differ from the Negative control. Data are presented as mean ± SD, and statistical significance is indicated by ns (not significant), * (p < 0.05), ** (p < 0.01), and **** (p < 0.0001).

The viability of HeLa cells treated with various formulations is illustrated in Fig. 8. The LNCs-Hydrogel composite showed a viability of ~ 90% (t = −0.8552, p = 0.4825, ns), with no statistically significant difference from the negative control, suggesting cytocompatibility. At the tested concentration of 50 μg/mL, none of the treatments reduced cell viability by more than 50%, except the positive control Emetine, a well-established inducer of apoptosis. This observation indicates minimal cytotoxicity of LNCs formulations compared to no treatment. Compounds with IC50 concentrations above 10 μg/mL are deemed safe candidates to use clinically^74–79^. Among the formulations, LNCs exhibited the highest viability (~ 135%, t = 16.1692, p = 0.0038, **), indicating enhanced metabolic activity. EFVLNCs and EFVLNCs Hydrogel composite exhibited viabilities of ~ 65% (t = −30.7915, p = 0.0011, **) and ~ 85% (t = −6.7239, p = 0.0214, *), respectively, indicating that the incorporation of EFV reduced cell viability, as expected for an active pharmaceutical ingredient. Emetine, used as a positive control, dramatically reduced cell viability to approximately 10% (t = −250.7546, p < 0.0001, ****), confirming its known cytotoxicity. These findings suggest that LNCs and the new LNCsHydrogel composite are cytocompatible, and the observed decrease in viability upon EFV loading aligns with the drug’s expected biological activity. The elevated viability observed with LNCs may reflect increased metabolic activity, potentially facilitated by mediumchain triglycerides (MCTs) as an energy source, soy lecithin supporting membrane integrity, and Tween 80 alleviating cellular stress^80–84^. Additionally, the aqueous medium and salt content likely help maintain osmolarity and hydration, contributing to an optimal environment for cell survival. Additionally, the resazurin assay likely detected this metabolic boost as increased viability. These results suggest that lipid-based systems, such as LNCs and their composites, can support high cell viability, warranting further investigation into their *invivo* application.

While preliminary cytotoxicity studies using HeLa cells have demonstrated promising biocompatibility of our formulations, follow-up experiments will be conducted on gastrointestinalrelevant cell lines to better represent oral conditions. Suitable cell lines for evaluating gastrointestinal cytotoxicity include Caco-2 (human intestinal epithelial cells), HT29-MTX (human colon carcinoma cells secreting mucus), and IEC-6 (normal rat small intestine epithelial cells)^85, 86^. These intestinal cell models are anticipated to provide critical insights into the safety, biocompatibility, and potential efficacy of our hydrogel- and lipid nanocapsulebased formulation for oral drug delivery, supporting further clinical translation.

### EFVLNCsCS/iCarp(AamcoAA) Hydrogel Composite as Oral Solid Dosage Form

For future studies, the EFVLNCCS/iCarp(AamcoAA) hydrogel composite can be optimized into a solid oral dosage form, enhancing patient convenience and therapeutic efficacy. Lyophilization could transform the composite into a dry, easily administrable powder or tablet, maintaining the integrity of both lipid nanocapsules and hydrogel components^87–89^. Freeze-drying would preserve the internal structure of the composite, minimizing aggregation or premature drug release. Additionally, compression into oral tablets or encapsulation in capsules could facilitate dosage uniformity, ease patient administration, and maintain sustained release properties^90,91^. Although solid dosage form development was not performed in this study, this approach represents a promising direction for enhancing the clinical applicability of EFV-loaded hybrid composites, potentially improving the oral bioavailability and therapeutic consistency of hydrophobic antiviral drugs.

## CONCLUSION

This study introduces an innovative lipid nanocapsule and hydrogel composite specifically developed for oral delivery of hydrophobic antiviral drugs. Biocompatible polysaccharides, chitosan (CS) and iotacarrageenan (iCar), were used to synthesize a porous hydrogel scaffold via free-radical copolymerization. The synthesized CS/iCarp(AamcoAA) hydrogel demonstrated high swelling capacity, facilitating effective encapsulation and sustained release of Efavirenzloaded lipid nanocapsules (LNCs). The composite improved EFV solubility and demonstrated controlled, pHresponsive release profiles under simulated gastrointestinal conditions. Furthermore, structural and cytotoxicity analyses confirmed the biocompatibility and safety of the developed formulation, suggesting favorable interactions within gastrointestinal environments. Future studies will focus on evaluating biocompatibility with relevant intestinal cell lines and optimizing mechanical and mucoadhesive properties. These next steps will be essential for translating this novel composite system into an effective clinical solution for not only orally delivering poorly soluble antiviral medications, but also for application in other mucosal tissues, such as vaginal drug delivery^92,93^, where hydrogels have been extensively employed as vehicles to deliver drugs embedded in nanoparticle, transdermal delivery and even pulmonary delivery^94^.

## Supplementary Material

Supplementary Files

**Solubility test**: the T-test in **Figure S1** shows that Efavirenz was significantly soluble in MCT oil unlike in water. **Hydrogel swelling capacity optimization: Table S1** lists all the hydrogels formulations prepared, with their corresponding swelling capacity values. **Table S2** shows the results of the analysis of variance of models fitted to the swelling capacity. **Potential mechanism of hydrogel formation**: **Figure S2** and **Figure S3** respectively describe the formation of free radical sites on chitosan and iota-carrageenan backbones under ammonium persulfate initiation, and the plausible reactional mechanism of CS/iCar-p(Aam-co-AA) hydrogel formation. **Standard error plot: Figure S4** presents the contour plot of the standard error at different points in the design space that shows small values of the standard error that ranged from 0.2 to 0.35, which is an indication that the means of the samples are a reliable reflection of the true population means. **Swelling capacity prediction: Table S3** lists automatically generated hydrogel composition batches for model optimization and confirmation.

This is a list of supplementary files associated with this preprint. Click to download.

- MukubwaManuscriptSupportinginformationJuly2025.docx

## Figures and Tables

**Table 1:** Ioptimal mixture design input variables

Low Limit		Constraint		High Limit
3.000	≤	A: IOTACARRAGEENAN	≤	6.000
3.000	≤	B: CHITOSAN	≤	6.000
40.000	≤	C: ACRYLIC ACID	≤	53.000
40.000	≤	D: ACRYLAMIDE	≤	53.000
1.000	≤	E: *N*,*N*METHYLENE BISACRYLAMIDE	≤	3.000
		**A + B + C + D + E**	=	**100.000**

**Table 2:** Observed swelling ratio mean against the prediction interval

	Response	Mean		95% Prediction	
		Predicted	Observed	95% PI low	95% PI high
Formulation 1	Swelling ratio	444.5	304.2	32.6	1407.1

**Table 3:** Comparison of the experimentally observed mean swelling ratio with the 95% prediction interval generated by the regression model.

	Response	Mean		95% Prediction	
		Predicted %	Observed %	95% PI low	95% PI high
Hydrogel + 58 mg/mL	**Encapsulation efficiency**	46.83	53.04	28.03	65.63
EFVLNCs					

## Data Availability

All data supporting the findings of this study are provided within the main article and its supplementary materials. Additional datasets generated and/or analyzed during the current study are available from the corresponding author upon reasonable request.

## Abbreviations

LNCs: Lipid Nanocapsules
CS: Chitosan
iCar: Iota-Carrageenan
EFV: Efavirenz
SEM: Scanning Electron Microscopy
EDS: Energy-Dispersive X-ray Spectroscopy
FTIR: Fourier Transform Infrared Spectroscopy
TGA: Thermal Gravimetric Analysis
DS: Droplet Size
PDI: Polydispersity Index
ZP: Zeta Potential
MBA: N,N’-Methylene Bisacrylamide
AA: Acrylic Acid
Aam: Acrylamide
SC: Swelling Capacity
SR: Swelling Ratio
IC50: Inhibitory Concentration 50%
ANOVA: Analysis of Variance
SLS: Sodium Lauryl Sulfate
DSC: Differential Scanning Calorimetry
